# Traditional Chinese Herbal Medicine Plays a Role in the Liver, Kidney, and Intestine to Ameliorate Hyperuricemia according to Experimental Studies

**DOI:** 10.1155/2021/4618352

**Published:** 2021-11-28

**Authors:** Li Xu, Li Li Lu, Jian Dong Gao

**Affiliations:** Department of Nephrology, Shuguang Hospital, Shanghai University of Traditional Chinese Medicine, TCM Institute of Kidney Disease, Shanghai University of Traditional Chinese Medicine, Key Laboratory of Liver and Kidney Diseases (Shanghai University of Traditional Chinese Medicine), Ministry of Education, Shanghai Key Laboratory of Traditional Chinese Clinical Medicine (20DZ2272200), Shanghai, China

## Abstract

In the last few decades, hyperuricemia has drawn increasing attention owing to its global prevalence. Observational surveys have manifested that there is a relation between hyperuricemia and increased risks of hypertension, chronic kidney disease, cardiovascular events, metabolic disorders, end stage renal disease, and mortality. As alternatives, Traditional Chinese medicinal herbs have demonstrated concrete effects in mitigating hyperuricemia in different experiments. Researchers have made efforts to investigate the role of herbal medicine in attenuating hyperuricemia. This review focuses on traditional Chinese herbal medicines that have been reported to ameliorate hyperuricemia in experimental studies.

## 1. Introduction

Hyperuricemia, by definition, is an elevated concentration of serum urate which is over the solubility threshold (approximately 6.8 mg/dl) [1]. There is a recently growing trend in hyperuricemia and gout prevalence worldwide according to epidemiological studies. In western populations, the prevalence is estimated to be approximately 21% for hyperuricemia and 1%–4% for gout according to population-based studies [2, 3]. According to the National Health and Nutrition Examination Survey (2007–2016), an investigation revealed a prevalence of 20.2% and 20.0% for hyperuricemia among men and women, respectively, from 2015 to 2016 in America and the incidence of hyperuricemia remained stable from 2007 to 2016 [4]. A nationwide cross-sectional investigation manifested that the prevalence rate of hyperuricemia was approximately 8.4% (9.9% in men and 7.0% in women) among Chinese adults from 2009 to 2010 [5]. According to study, the pooled prevalence rate for hyperuricemia in Mainland China, from 2000 to 2014, was approximately 13.3% [6]. Clinical surveys have shown that hyperuricemia serves as an independent risk factor for cardiovascular events, chronic renal failure, diabetes mellitus, and metabolic disorders [7, 8].

Urate, the end-product, in human, of the purine metabolism in liver hepatocytes, excretes via the liver or go through the blood and excretes via kidney or the intestines. In general, exogenous factors for hyperuricemia can roughly be attributed to high intake of dietary purine, while endogenous causes are generally ascribed to enhanced uric acid production and a decreased uric acid excretion. Damaged excretion of uric acid is due to abnormality in kidney urate transporter, while increased uric acid production is because of an excess of active XOD [9].

Urate-lowering therapy is currently the major medicinal therapy for hyperuricemia. Urate-lowering agents can be classified based on different mechanism of effects: xanthine oxidase inhibitors such as allopurinol and febuxostat via lowering production of urate; uricosuric drugs such as benzbromarone and probenecid, increasing kidney urate excretion through inhibiting its reabsorption; and recombinant uricase inhibitors which enable degrade of uric acid [8]. Currently, there are various alternatives such as traditional Chinese medicine (TCM), as a supplement for treatment for hyperuricemia. Experiments have manifested that TCM is able to mitigate hyperuricemia in various murine models, by inhibiting XOD activity, modulating urate transporters, and exerting anti-inflammation and antioxidation activity.

To our knowledge, there are some review articles concerning hyperuricemia-associated disease, such as a systematic review and meta-analysis of clinical studies about TCM treatment of hyperuricemia [10], a systematic review about TCM treatment in lowering uric acid in both clinical and experimental surveys [11] and a review about TCM treatment of gout in both clinical and experimental studies [12]. Furthermore, to be more specific in another way on actions and mechanisms of TCM in mitigating hyperuricemia, this review will be divided into several parts based on the different actions of herbs, including inhibition of XOD effects (mainly in the liver or serum), regulation of urate transporters in the kidney or urate transporters in the intestine, antioxidant, and anti-inflammatory effects (Table 1). However, these effects are not strictly independent of each other; in fact, some herbs do not exert just one single effect. These experimental researches may pave way for future clinical use.

## 2. Herbs That Reduce Uric Acid by Inhibiting Its Production

Herbs that show hepatic effects (mainly effects of XOD inhibitors) are as follows.

XOD, mainly found in the liver, spleen, serum, lung, kidney and intestinal epithelium, and milk of mammals (this review mainly talks about liver and serum XOD), is important in the production of uric acid. XOD catalyzes oxidation process of hypoxanthine to xanthine and finally to uric acid.

### 2.1. Herbs in Simples

*Cinnamomum cassia*/*Cinnamomum cassia* (L.) J. Presl, a major herb in many prescriptions, has long been utilized to treat hyperuricemia and gout. Experiments have shown [13] that *Cinnamomum cassia* (L.) J. Presl possesses various biological activities in mitigating uric acid related disorders. Kong et al. [14] concluded that the methanol extracts of this herb exert inhibitory effects on bovine milk XOD in their previous study. In their later study [13], it was reported that the methanol extract of the stem bark of this herb, called Cassia oil, significantly decreased serum as well as liver urate levels in oxonate-induced hyperuricemia mice. Meanwhile, they found that Cassia oil was equal potent as allopurinol at a dose of 600 mg/kg. Cassia oil markedly lowered xanthine dehydrogenase/oxidase activities in the mouse liver, with an apparent time and dose dependence in hyperuricemic mice, which could explain, at least partly, the hypouricemic effects [13].

*Cinnamomum osmophloeum* Kaneh. leaf essential oil demonstrates plenty of biological activities, such as anti-inflammatory activities. A study in Taiwan [15] found that the *C. osmophloeum* leaves' essential oil exhibited the most potent inhibitory effects on XOD compared to ethanolic and hot water extracts. No marked XOD inhibitory effects were observed in these two extracts. In addition, researchers found that cinnamaldehyde exhibited potent inhibitory effects against XOD compared to the other main compounds of *C. osmophloeum* leaves essential oil. Subsequently, cinnamaldehyde was further investigated and found to markedly decrease uric acid levels in potassium oxonate-induced mice. Impressively, the constituents of *Cinnamomum osmophloeum* Kaneh. leaf essential oil are similar to those of *Cinnamomum cassia*. Indeed, the inhibition of XOD can reduce the production of uric acid, so this ability to lower uric acid is, at least partly, achieved by XOD inhibition activity.

The extracts of *Lonicera hypoglauca* Miq. were reported to have the ability to suppress XOD, which was examined in a recent study [16]. Both the ethanol extract of the *Lonicera hypoglauca* Miq. leaves (LH-crude) and its derived EtOAc-soluble subfractions (LH-EA) exerted significant inhibitory effects on XOD activity. In addition, in potassium oxonate induced hyperuricemic mouse model, LH-EA decreased serum urate (sUA) levels. Finally, researchers isolated a novel bisflavonoid, loniceraflavone, which significantly inhibited XOD. Thus, loniceraflavone might be the main active component.

Curcumin, derived from the rhizomes of *Curcuma longa* L., is a natural hydrophobic polyphenol. In the treatment of hyperuricemia, it has been utilized as an anti-inflammatory and antioxidant drug and seems to be efficacious [17]. Recent surveys have shown that it decreases uric acid levels via suppressing XOD in serum and in the liver in potassium oxonate-induced hyperuricemia mice and also has nephroprotective effects by suppressing the nucleotide-binding oligomerization domain, leucine-rich repeat-containing proteins (NLR) family pyrin domain containing 3 inflammasome (NLRP3) in hyperuricemic mice [17].

*Smilax china* L. showed hypouricemic and renoprotective effects in hyperuricemic animals. In hyperuricemic mice, the ethyl acetate fraction (EAF) of *Smilax china* L. is stronger in attenuating urate levels than the other four fractions (chloroform, n-butanol petroleum ether, and residual ethanol fraction) [18]. The EAF markedly reversed the elevated serum creatinine (Scr) levels and blood urea nitrogen (BUN) levels to normal. In addition, the EAF prevented tubular and interstitial impairment effectively in the renal histopathological sections of hyperuricemic rats induced by fructose [18]. This study concluded that the hypouricemic activity of the EAF is due to the decreased uric acid production via suppression on XOD activity and the enhanced urate excretion via increase in urinary volume and fraction excretion of uric acid (FEUA) [18]. Furthermore, this study found that nine compounds derived from the EAF exhibited distinct inhibitory activities against XOD [18]. From the weakest through strongest were engeletin, astilbin, polydatin, chlorogenic acid, protocatechuic aldehyde, caffeic acid, rutin, oxyresveratrol, and resveratrol. All, except engeletin, possessed a certain activity, and only engeletin, polydatin, chlorogenic acid, caffeic acid, rutin, oxyresveratrol, and resveratrol exerted competitive inhibition against XOD [18]. They showed in vitro different XOD inhibitory activities, which may explain the hypouricemic effect of EAF [18]. Of note, to testify the effect of astilbin, in another study [19], researchers fed fructose-induced hyperuricemic rats with different dosages of astilbin (1.25, 2.5, and 5.0 mg/kg). They drew that astilbin significantly reduced the serum uric acid level through increasing the urinary uric acid level and fractional excretion of urate but not suppressing the XOD activity, which is in accordance with the former study. Additionally, serum creatinine and blood urea nitrogen were recovered in these rats. They conducted further investigation and suggested that astilbin could prevent the renal damage through inhibiting the expression of transforming growth factor-*β*1 (TGF-*β*1) and connective tissue growth factor (CTGF) and showed a renoprotective action through suppressing formation of monosodium urate and production of prostaglandin E2 (PGE2) and interleukin-1 (IL-1). The renoprotective effects may make *Smilax china* L. more applicable in clinics with the knowledge of many side effects caused by allopurinol or febuxostat; however, this needs to be further verified.

Aurantii fructus immaturus, named Zhi Shi in Chinese, is derived from citrus plants and has long been widely utilized in China. Carbon dots are derived from charcoal used in TCM. Carbon dots derived from Aurantii fructus immaturus carbonisata, a nanomaterial, has been shown to decrease sUA levels via suppression on XOD activity in vitro and in hyperuricemic rats [20].

*Cordyceps militaris* (L. ex Fr.) Link is a kind of Ascomycetes (a fungus). In potassium oxonate-induced hyperuricemic mice model, the ability of exopolysaccharides produced by *Cordyceps militaris* (L. ex Fr.) Link (EPCM) to ameliorate hyperuricemia was tested. In hyperuricemic mice, compared to allopurinol, EPCM (400 mg/kg) exerted equal effects on serum uric acid levels, BUN levels, and liver XOD activity. The antihyperuricemic effect of EPCM is due to a reduction in urate production and XOD inhibitory activities. The renal protection of EPCM, compared to allopurinol, was also demonstrated in this study [21].

### 2.2. Herbs in Formulas

Ermiao wan, which is composed of *Phellodendron chinense* Schneid. (Rutaceae)/phellodendri cortex (15 g), and rhizome atractylodis/*Atractylodes lancea* (Thunb.) DC (Asteraceae)/(15 g), is widely utilized in China for hyperuricemia and gout treatment. Ermiao wan has shown antihyperuricemic action in normal mice and hyperuricemic mice as well in vivo [22]. This study demonstrated that the phellodendri cortex was more potent in lowering sUA than Ermiao wan, while atractylodis rhizome did not show any such effect but could strengthen the effect of phellodendri cortex. Therefore, the antihyperuricemic activity of Ermiao wan and rhizome atractylodis in mice with hyperuricemia was partly achieved through other mechanisms, instead of xanthine dehydrogenase and XOD suppression in vivo [22]. Ermiao wan was further investigated recently by biochemical and histopathological assays, which confirmed that Ermiaowan markedly reduced uric acid levels and notably improved fibrosis problems in the kidney [59].

## 3. Herbs That Reduce Uric Acid by Enhancing Its Excretion

Uric acid excretion is handled primarily by urate transporters of the kidney or intestine. For the kidney, the handling of uric acid can be generally separated into four steps: glomerular filtration, tubular reabsorption, tubular secretion, and reabsorption after tubular secretion. The whole process is regulated via urate transporters (reabsorptive and secretory) in the proximal tubule in the kidney, including reabsorptive urate exchanger (URAT1), glucose transporter (GLUT9), organic anion transporter (OAT1), and secretory exchange transporter (ATP-binding cassette subfamily G member 2, ABCG2). In the intestine, there is also a secretory exchange transporter (ABCG2), which can also assist the excretion of uric acid.

Some herbs can exert renal actions (renal urate transporters).

### 3.1. Herbs in Simples

*Cordyceps militaris* (L. ex Fr.) Link. in traditional Chinese books is able to influence serum uric acid levels. As EPCM was discussed in “liver XOD inhibition” [21], researchers found that the water extract of *Cordyceps militaris* exhibits antihyperuricemic actions, and the possible mechanism is through the inhibition activity on URAT1 protein levels. The water extract of *Cordyceps militaris* exhibits excellent hypouricemic activity, and it has no adverse impact on liver, spleen, and renal functions [23]. In hyperuricemic mice, WECM, at doses of 200, 100, and 50 mg/kg, showed a significant reduction in serum uric acid levels of 162, 184, and 189 *μ*mol/L, respectively (*P* < 0.01). The pivotal functional residues might be LYS145, ARG477, ARG325, and ASP168 according to molecular docking examinations. Four compounds were identified by virtual screening of *C. militaris* as a potential active ingredient [23]. These investigations suggest that *C. militaris* may ameliorate hyperuricemia in multiple ways, which need to be further investigated.

*Herbaceous peony*/*Paeonia lactiflora* Pall. flowers were utilized in diet therapy in many nations including China. Previous reports have suggested that herbaceous peony flowers have antioxidant activity. Total glucosides are derived from herbaceous peony root and are widely utilized clinically to treat gouty conditions. It was reported to show effectiveness in lowering uric acid levels [24]. To test this effect, the total glucosides of herbaceous peony flower (TGPF) was examined in vivo. TGPF reduced blood TNF-*α*, MCP-1, UA, Cr, BUN and, XOD and improved weight loss in rats with urate nephropathy [24]. Moreover, TGPF upregulated renal OAT1, downregulated renal GLUT9 and URAT1, and improved in the spleen, kidney, and thymus histopathological alterations [24]. It diminishes serum uric acid levels, which is mainly achieved by regulating urate transporters and mitigate kidney pathology related to hyperuricemia [24]. In conclusion, TGPF is a promising therapeutic agent against hyperuricemia.

Rhizoma Dioscoreae Nipponicae, which is derived from *Dioscorea nipponica* Makino, is a commonly utilized traditional Chinese medicinal herb in treatment of arthrodynia, arthritis, and arthroncus. Total saponins of Rhizoma Dioscoreae Nipponicae reversed changes induced by potassium oxonate effectively in mouse renal OAT1, OAT3, URAT1, and GLUT9 mRNA and protein levels, leading to an increase in kidney urate excretion in potassium oxonate-induced mice [25]. In conclusion, these outcomes suggest that the Rhizoma Dioscoreae Nipponicae total saponins exhibited in hyperuricemic animals a uricosuric action on regulation of the kidney organic ion transporters [25].

Fraxini Cortex/*Fraxinus chinensis* subsp. *rhynchophylla* (Hance) A.E.Murray, also known as Qinpi in TCM, has been utilized to treat hyperuricemia and has antioxidant, anti-inflammatory, and diuretic effects. Clinically, Qinpi from different regions exert significantly different therapeutic effects. Zhou et al. [26] demonstrated that Fraxini cortex extracts from different regions could mitigate hyperuricemia by markedly decreasing the serum uric acid, BUN, and serum creatinine levels and significantly enhancing urine creatinine and urine uric acid levels. In addition, it could also improve renal pathological alterations. Under treatment with Fraxini cortex extracts, the mRNA and protein levels of both GLUT9 and URAT1 were downregulated. Shaanxi Qinpi extracts showed preferable effect on alleviating hyperuricemia and regulating GLUT9 and URAT1 compared to the extracts of Henan Qinpi and extracts of Hebei Qinpi. High Performance Liquid Chromatography analysis manifested that the percentage of four ingredient, aesculetin, aesculin, fraxetin, and fraxin may be a pivotal factor for the effectiveness of Qinpi [26]. In China, geo-authentic crude drugs and genuine traditional Chinese drugs have better quality and curative effects than normal drugs. This study proved that this is true for Shaanxi Qinpi, and Shaanxi Qinpi is what we consider as a geo-authentic crude drug in TCM. However, further investigation is required to elucidate the mechanism.

### 3.2. Herbs in Formulas

The Simiao pill, which consists of phellodendri cortex/*Phellodendron amurense* Rupr. (120 g), atractylodis rhizome/*Atractylodes lancea* (Thunb.) DC (60 g), Achyranthes root/*Achyranthes bidentata* Blume. (60 g), and coix seed/*Coix lacryma-jobi* L. var mayuen (Roman.) Stapf (120 g), is a very frequently used prescription in TCM and can be utilized to treat hyperuricemia and gout. In hyperuricemic mice, Simiao pill markedly increased FEUA levels and decreased serum uric acid levels dose-dependently [27]. Moreover, it reversed oxonate-induced alterations effectively in the mRNA and protein of transporters in the kidney, including renal mOAT1, mURAT1, and mGLUT9, leading to an increase in kidney urate excretion in hyperuricemic mice. Furthermore, Simiao pill reduced sCr levels and enhanced mRNA and protein levels of renal mOCTN1, mOCTN2, and mOCT1, mOCT2, which contributes to an improved renal function in this mice model. These results indicate that in hyperuricemic animals, the Simiao pill possesses uricosuric and kidney protective effects via regulation of kidney organic ion transporters [27].

Wuling San, which is composed of *Polyporus umbellatus* (Pers.) Fries (180 g), *Wolfiporia cocos* (Schw.) Ryv. & Gibn (180 g), *Alisma plantago-aquatica* subsp. orientale (Sam.) Sam. (300 g), Cinnamomi Cortex/*Neolitsea cassia* (L.). Kosterm. (180 g), and *Atractylodes macrocephala* Koidz. (180 g), is a famous prescription in TCM. It promotes kidney function and diuresis. In a study [28] in 2013, Wuling San effectively decreased serum UA and serum Cr levels and increased 24 h urate and creatinine excretion, as well as the FEUA in hyperuricemic mice induced by potassium oxonate. Thus, Wuling San exhibits the capacity of enhancing excretion of urate and improving kidney function. The mechanism was investigated in the same study in which Wuling San downregulated renal mRNA and protein levels of mGLUT9 and mURAT1 and upregulated those of mOAT1 in mice with hyperuricemia. Furthermore, in this model, from the perspective of kidney protection, Wuling San upregulated kidney mRNA and protein levels of mOCT1, mOCT2, and mOCTN2. In a study [29] in 2015, in hyperuricemic mice induced by high fructose, urate-lowering and nephroprotective effects were confirmed. Wuling San markedly decreased levels of serum uric acid, blood urea nitrogen, and creatinine and enhanced FEUA in fructose-induced hyperuricemic mice. It helped reverse the fructose induced dysregulation of URAT1, GLUT9, ABCG2, and OAT1, as well as OCT1 and OCT2 in fructose-fed mice. Wuling San mitigated the inflammatory cells infiltration in the kidney glomerulus and exerted inhibitory effects on TLR4/MyD88 signaling activation, thus inhibiting nuclear factor *κ*B signaling and activation of mitogen-activated protein kinases. Furthermore, Wuling San, in fructose-fed hyperuricemic mice, suppressed the activation of NLRP3 inflammasome and thus reduced renal secretion of IL-1*β*. In high fructose-fed hyperuricemic mice model, Wuling San modulating kidney organic ion transporters and thus reducing serum uric acid levels might explain the inhibition of inflammatory reaction. The molecular mechanism might be related to the suppression of TLR4/MyD88 signaling and activation of NLRP3 inflammasome to decrease IL-1*β* excretion [29].

The Erding formula (EF) is recorded in the Chinese Pharmacopeia, which consists of *Viola yedoensis* Makino/*Viola philippica* Cav., *Lobelia chinensis* Lour, *Taraxacum mongolicum* Hand.-Mazz., and *Isatis indigotica* Fort. In 2018, the aqueous extracts of EF which contain 24 g raw materials/kg (each herb is 6 g raw materials/kg) were investigated in a study [30]. This study found that the EF can enhance UA excretion. Moreover, *Viola yedoensis* Makino was the only herb that exhibits hypouricemic effect, while all four herbs of the EF had analgesic and anti-inflammatory effects. The urate-lowering effect might be achieved by suppressing mRNA expression of URAT1 and enhancing that of OAT3. This study provides pharmacological views into the part of the formula and single herbs in uric acid excretion. Erding granule (EDG) is the granular form of EF. To compare the efficacy of each EDG extracts on concentration of serum uric acid in mice with hyperuricemia, a study [31] in 2019 tested 95% ethanol, 50% ethanol, and water extracts of EDG. The study manifested that 50% ethanol extract was more potent in decreasing uric acid levels than the water extract and 90% ethanol extract. They further examined this effect of EDG-50 and found that it was mainly achieved through downregulation of GLUT9 and URAT1 protein expression and upregulation of OAT1 protein expression. Specifically, they tested these three extracts and found that although the active compounds remain unclear, the constituents of EDG-50 extract were distinct from those of EDG-95 and EDG-S extracts. Thus, they inferred that the hypouricemic effect may attribute to the different constituents, including alkaloids, and flavonoids, organic acids, which require further investigation. Moreover, they found that the EDG is nephroprotective; however, further research is also needed on the pharmacodynamics of EDG prescription drugs. Overall, the EF (including Erding granule) is efficacious in lowering uric acid levels in mouse models, and this effect is mainly achieved by regulating urate transporters.

Compound Tufuling granules consist of 35g of rhizoma smilacis glabrae/*Smilax glabra* Roxb., 18 g of Rhizoma Dioscoreae Hypoglaucae/*Dioscorea tokoro* Makino ex Miyabe, 15 g of Pseudobulbus Cremastrae seu Pleiones/*Cremastra appendiculata* (D. Don) Makino, 10 g of Semen Vaccariae/*Gypsophila vaccaria* (L.) Sm, and 10 g of Radix Achyranthis Bidentatae/*Achyranthes bidentata* Blume. [32]. It is used in clinical conditions such as gout and hyperuricemia because it exerts anti-inflammatory and analgesic effects [32]. It was found to have a urate-lowering effect in earlier experiments, and this effect was later confirmed in a recent study that it reduced serum uric acid and enhanced the FEUA by inhibiting glucose transporter 9 in hyperuricemic mice in vivo [32]. This effect was further clarified in another study [33] that compound Tufuling granules could lower serum uric acid levels and protect kidney function, and they are anti-inflammatory.

Xie-Zhuo-Chu-Bi-Fang, which is utilized clinically in a Guangzhou Hospital, is composed of *Smilax glabra* Roxb., rhizome (Tufuling, smilacis glabrae rhizoma) (35 g), *Heterosmilax japonica* Kunth, rhizome (Bixie, Heterosmilacis Rhizoma) (18 g), *Cremastra appendiculata* (D. Don) Makino, rhizome (Shancigu, Cremastrae Pseudobulbus) (15 g), *Achyranthes bidentata* Blume., root (Niuxi, Achyranthis Bidentatae Radix) (10 g), and *Vaccaria hispanica* (Mill.) Rauschert, seed/*Gypsophila vaccaria* (L.) Sm (Wangbuliuxing, Vaccariae Semen) (10 g). In basic research, previous study [34] showed that Xie-Zhuo-Chu-Bi-Fang could effectively alleviate hyperuricemia in mice model. Later, they further investigated this formula and concluded that the ability to enhance uric acid excretion and reduce its level was mediated by upregulation of miR-34a to inhibit URAT1 mRNA expression [35].

The Quzhuotongbi decoction (QZTBD) is composed of Smilax glabra Roxb./Glabrous Greenbrier Rhizome) (60 g), *Dioscorea septemloba* Thunb. (30 g), *Zea mays* L./Maydis stigma (15 g), *Coix lacryma-jobi* var. ma-yuen (Rom. Caill.) Stapf/coix seed (30 g), *Alisma plantago-aquatica* Linn./Alismatis rhizome (15 g), *Humulus scandens* (Lour.) Merr./*Humulus scandens* (15 g), *Taxillus sutchuenensis* (Lecomte) Danser/Parasitic loranthus (15 g), *Siegesbeckia orientalis* L./Herba Siegesbeckiae (18 g), *Curcuma longa* L./turmeric (12 g), *Corydalis yanhusuo* (Y.H.Chou & Chun C.Hsu) W.T.Wang ex Z.Y.Su & C.Y.Wu/Corydalis Rhizoma (18 g), and *Citrus medica* Linn./Citrus medica (12 g). QZTBD is a formula prescribed to hyperuricemia, without serious adverse effects reported. Chen et al. [36] mentioned that a previous Chinese study showed its clinical urate-lowering effects, which was published only in a TCM journal. Chen et al. then performed a basic research to test the effect in mice. Based on method of gas chromatography-mass spectrometry on serum metabolomics, they noticed great metabolic disorders in a high yeast-fed rat model, involving abnormalities in glycolytic pathway and nucleotide and amino acid metabolism. After feeding with allopurinol and the distilled extract of QZTBD, serum UA levels were efficiently reduced. Other metabolites, such as glutamine and aspartate, which participate in purine metabolism, went back to normal levels. However, allopurinol and QZTBD exerted different effects on metabolites such as pyroglutamate, glutamate, xanthine, and galactonate, suggesting that these two drugs function in distinct ways to reduce sUA levels [36]. They mentioned in their study that recent research showed that the urate-lowering effects of QZTB were mainly achieved by enhancing urate excretion. They speculated that this may have something to do with the inhibition of URAT1 expression. To confirm this, further investigations are necessarily required in the future.

### 3.3. Herbs That Exert Renal and Intestinal Actions (Renal and Intestinal Urate Transporters)

#### 3.3.1. Herbs in Simples

Dioscin, the rhizoma of *Dioscorea septemloba* (Diocoreacea)/*Dioscorea tokoro* Makino ex Miyabe, is utilized for rheumatism, joint pain, and diuresis. The action of decreasing uric acid as well as increasing urate excretion was demonstrated in hyperuricemic mice. In vivo, the dioscin metabolite, tigogenin, was considered as an active constituent with antihyperuricemic effect. The hypouricemic effects are at least partially achieved by inhibiting URAT1 and promoting ABCG2 [37].

*Cichorium intybus* L., recognized in common as chicory, comes from the Asteraceae family. A study found that there was a close relationship between the hypouricemic activities of chicory and the main effective compounds, aesculin, chicoric acid, chlorogenic acid, 14(15)-diene-3*α*-ol, 13,14-seco-stigma5(6), and isochlorogenic acid A/B/C, [38]. In a previous study, it showed potent antihyperuricemic activities associated with reducing hepatic uric acid production through downregulating XOD activity and enhancing uric acid excretion through upregulating the mRNA expression of renal OAT3. In a study [39] in 2017, the effect of extra-renal uric acid excretion and the possible underlying mechanism was investigated. Chicory promotes uric acid excretion by the intestine, possibly associated with the increase of intestinal uric acid excretion by downregulating the mRNA and protein expression of ABCG2. In a study [40] in 2018, the chicory extract was further investigated both in vivo and in vitro. Researchers found that the chicory extract reduced serum uric acid levels, and its activity on postponing renal damage progression was better than that of benzbromarone in rats with hyperuricemia. In vitro, the suppression of GLUT9 protein expression in kidney tubules was observed, which is the main factor in action. Moreover, the decrease in serum uric acid concentrations may explain why renal damage was alleviated. This indicates that chicory is a good alternative when renal dysfunction occurs in hyperuricemia.

## 4. Herbs That Reduce Uric Acid by Inhibiting Its Production and Enhancing Its Excretion

### 4.1. Herbs That Show Hepatic and Renal Dual Actions

#### 4.1.1. Herbs in Simples

*Smilax glabra* Roxb. (SG), “Tufuling,” in TCM, is used to treat conditions akin to hyperuricemia and gout. To verify the hypouricemic effect of *S. glabra* and determine the active compounds, total flavonoids of *S. glabra*, involving neoastilbin, astilbin, neoisoastilbin, and isoastilbin, were obtained in a previous study [41]. The study suggested in hyperuricemic mice that the total flavonoids of *Smilax glabra* Roxb. have potent hypouricemic effects via suppressing actions of XOD and upregulating the expression of OAT1, organic cation/carnitine transporter 2(OCTN2), and the mRNA (mOAT1 and mOCTN2) in kidney tissue. *S. glabra* Roxb. also exhibits antioxidative ability, which will be explicated in the “antioxidative” part of this study.

*Gnaphalium pensylvanicum* willd. is used in China to treat rheumatoid arthritis, cough, and inflammation. *G. pensylvanicum* extract reduced the uric acid effect by downregulating GLUT9 and URAT1 expression, upregulating OAT1 expression, and inhibiting XOD activity in hyperuricemic mice induced by potassium oxonate. Additionally, *G. pensylvanicum* extracts proved to be renoprotective. Caffeoylquinic acid derivatives, the main active components of G. pensylvanicum extracts, may partially explain the uricosuric or XOD inhibition effects [42].

*Smilax riparia*/*Smilax riparia A*. DC is usually used in symptomatic treatment of gout and is reported to be effective. One study demonstrated that *S. riparia* could enhance uric acid excretion due to downregulation of renal mURAT1 by *Smilax riparia* total saponins [43]. The total saponins of this herb were for first time ever, shown to exhibit potent uricosuric activities in mice with hyperuricemia by downregulating kidney mURAT1 [43]. Subsequently, in a second study [44], the researchers isolated two steroidal glycosides from the total saponins of *Smilax riparia* A. DC roots, named smilaxchinoside A and smilaxchinoside C. They found that these two constituents exhibit great uricosuric effects. Meanwhile, the inhibition of XOD and the reduction in renal mURAT1 were also observed, which led to an enhancement in uric acid excretion as well as a decrease in kidney dysfunction induced by hyperuricemia [44]. In a third study [45], they found two other steroidal glycosides, named timosaponin J and riparoside B. Both exhibit great uricosuric effects in mice with hyperuricemia, and the effect is mainly achieved by reducing kidney mURAT1 and suppressing XOD action to some degree, which help increase uric acid excretion as well as attenuation of hyperuricemia-induced disorder in kidney [45]. In addition, *S. riparia* exhibited synergistic effects with allopurinol. A later study proved that *S. riparia* can improve allopurinol's actions in vivo by reducing serum uric acid levels [46].

Morin is derived from the twigs of *Morus alba* L. and was recorded to treat gout in TCM literature. It has been proven to possess a strong suppressing effect on renal reabsorption of urate in rats. Urate uptake was observed in the brush border membrane of the renal proximal tubule, where there is a urate-anion transporter (URAT1) that is mainly responsible for the uricosuric effect in rats. Therefore, URAT1 has a lot to do with the effects in this study, but the regulation of URAT1 needs to be further investigated. In addition, morin demonstrated inhibitory effects on XOD. The dual actions of morin, uricosuric effect, and XOD inhibitory activity could at least partly explain why morin could lower uric acid levels [47].

*Paederia scandens* (Lour.) Merrill (Rubiaceae), which has long been used in TCM, consists of flavonoids, iridoids, triterpenes, sterols, and essential oils. *P. scandens* is a medicinal herb applied in conditions akin to gout in TCM. In an investigation [48], the extract of *P. scandens* was shown to be a potent inhibitor of XOD and has inhibitory effects on renal urate reabsorption. The dual effects of this ingredient account for its hypouricemic effect in vivo. Notably, the serum uric level was lowered not in normal mice, but only in mice with hyperuricemia. The antihyperuricemic effect of allopurinol was seen in both groups. Indeed, elevated serum uric acid levels beyond the threshold could possibly induce many pathological conditions, including gout. However, from a different perspective, uric acid is an antioxidant and is not harmful in any case. The uricase gene missing in humans can be for evolutionary benefits, such as a longer lifespan. This additional advantage of this compound may be more suitable in this regard as a hypouricemic agent [48].

Green tea polyphenols/*Camellia sinensis* (L.) Kuntze (GTPs) are the major active fractions in green tea with various pharmacological effects. A previous study demonstrated that GTPs can decrease serum uric acid levels in mice with PO-induced hyperuricemia via two pathways. First, GTP markedly decreased the expression of XOD in the liver and its activity in the serum as well as liver, thus inhibiting UA production. Second, GTP substantially decreased expression of URAT1 and promoted expression of OAT1 and OAT3 and accordingly increased secretion of urate and suppressed reabsorption of urate [49].

The combination of Alismatis Rhizoma/*Alisma plantago-aquatica* L. and rhizoma smilacis glabrae/*Smilax glabra* Roxb. (RSG) has been used for its urate-lowering effect. A study confirmed in hyperuricemic rats that Alismatis Rhizoma/*Alisma plantago-aquatica* L. and RSG alone or in combination are capable of lowering serum uric acid levels, with a more marked effect in the combination groups. This mechanism might have something to do with its suppression on XOD activity, improvement of kidney damage, and downregulation of URAT1 expression in the kidney [50].

#### 4.1.2. Herbs in Formulas

Sanmiao wan, a common formulation in TCM, is widely utilized to treat hyperuricemia and gout. Sanmiao wan, actually Ermiao wan added to achyranthes root/*Achyranthes bidentata* Blume., consists of the achyranthes root, phellodendri cortex, and atractylodes rhizome. Sanmiao wan exhibits dual actions in lowering uric acid by suppression on hepatic XOD action to inhibit uric acid production and by downregulating kidney mURAT1 to promote excretion of urate and reduce reabsorption of urate in mice with hyperuricemia [51].

The Chuanhu anti-gout mixture was previously demonstrated to be noninferior to colchicine in treating acute gouty arthritis [52]. It is comprised of 15 g of *D. nipponica*/*Dioscorea nipponica* Makino, 15 g of P. cuspidatum/*Reynoutria japonica* Houtt., 30 g of honeysuckle rattan/*Lonicera japonica* Thunb., 15 g of Radix cyathulae/*Cyathula officinalis* K.C. Kuan, 15 g of glabrous greenbrier/*Smilax glabra* Roxb., 15 g of Radix saposhnikoviae/*Saposhnikovia divaricata* (Turcz. ex Ledeb.) Schischk., 15 g of Radix clematidis/Clematis chinensis Osbeck, 15 g of Rhizoma chuanxiong/*Conioselinum anthriscoides* “Chuanxiong,” 15 g of coix seed/*Coix lacryma-jobi* var. ma-yuen (Rom.Caill.) Stapf, 6 g of Glycyrrhizae radix et rhizome/*Glycyrrhiza* uralensis Fisch. ex DC., in addition, 2 g of sodium alginate was added. In a later study, for convenience and patient compliance, the researchers developed a granule formulation and slightly modified formulation by adding cassia twig/*Neolitsea cassia* (L.) Kosterm. (10 g). Borneol (0.3 g) was added to the original one [52]. The original Chuanhu anti-gout mixture and its modified formula markedly decreased levels of sUA in mice with hyperuricemia, which could be partly attributed to the decrease in liver XOD and kidney URAT1 levels [52]. In addition, both could reduce creatine levels in hyperuricemia mice.

The ShiZhiFang (SZF), a TCM formula which consists of plantago seeds/*Plantago asiatica* L. (60 g), white mustard seeds/*Sinapis alba* L. (30 g), vaccaria seeds/*Gypsophila vaccaria* (L.) Sm 30 g, and abutilon seeds/*Malva verticillata* L. (30 g), is a compound that our research group usually applied to the treatment of hyperuricemia and hyperuricemia-related diseases, such as urate nephropathy. The clinical efficacy and safety of lowering uric acid levels were confirmed in our previous clinical study [53]. In our later study to test the efficacy and mechanism of this formula upon metabolism of uric acid, we found that SZF may be involved in facilitating the transcription as well as expression of renal rOAT3 and rOAT1 and inhibiting serum XOD activity [53].

### 4.2. Herbs That Show Hepatic, Renal, and Intestinal Triple Actions

#### 4.2.1. Herbs in Simples

Dendrobium officinalis six nostrum (DOS), the transformation of the TCM nostrum SiMiao Wan by adding *Dendrobium officinalis*/Dendrobium officinale Kimura & Migo (the specific dose was not seen in this article), is in accordance with the theory of TCM. In a recent study [54], DOS was shown to enhance UA excretion and decrease UA production in a rat model of hyperuricemia with hyperlipidemia. This study suggests that DOS might decrease UA production through upregulating the hypoxanthine-guanine phosphoribosyl transferase (an enzyme which catalyzes hypoxanthine and guanosine monophosphate to inosine monophosphate) and through inhibiting XOD in the liver. DOS can enhance excretion by increasing the content of renal and intestinal ABCG2 and reducing the content of PDZK1 (which has the opposite effect on serum uric acid compared to that of ABCG2 in the kidney). In addition, DOS can also improve renal and intestinal pathological alterations by decreasing the activation of NLRP3 and caspase 1 inflammatory bodies. Of note, we can only see the upregulation of HPRT1, whether DOS can enhance the activation of HPRT1 remains unclear.

## 5. Herbs That Exhibit Antioxidative and Anti-Inflammatory Effects

Urate-lowering therapy is the main, but not the only, drug therapy for hyperuricemia. Long-term hyperuricemia conditions intrinsically include pathological changes, such as oxidation and inflammation. Through activating complex cascades of proinflammation that impair cells and tissues, elevated uric acid levels beyond the threshold enhance the expression of inflammatory proteins [60]. Oxidative stress plays a pivotal part in the pathophysiology of kidney damage. Investigations [61, 62] have confirmed that UA-induced oxidative stress, for inflammatory reactions, is similar to a second messenger. In earlier surveys, uric acid was regarded as an antioxidant protecting the organism from impairment brought by oxidative stress [63]. Nevertheless, investigations after have proven that excessive uric acid induce much more impairment to endothelial cells than being an antioxidant [64, 65]. UA may accumulate in the kidney due to the absence of urate oxidase, leading to renal inflammation. Except for inducing inflammation, uric acid also plays a vital role in inducing intrarenal vasoconstriction and renal damage [66].

### 5.1. Herbs in Simples

Rhizoma smilacis glabrae/*Smilax glabra* Roxb. (RSG), the rhizome of the Liliaceae plant, is called Tufuling in TCM. RSG water extract showed potent capacity to alleviate hyperuricemia and gout in murine model induced by monosodium urate and potassium oxonate [55]. Hong et al. demonstrated in their previous study [56] that uric acid was able to activate the production of the reactive oxygen species, afterward leading to endothelial dysfunction. In their later in vitro experiments [57], they found that RSG could alleviate the hyperuricemia induced oxidative stress through upregulating the expression of catalase.

Galangin belongs to the flavonol class of flavonoids and is obtained from *Alpinia officinarum* Hance, a traditional Chinese medicinal plant. Galangin has been used as an antioxidant and anti-inflammatory agent. Lu et al. found that galangin can effectively improve nephric inflammation through inhibiting inflammatory reactions, which may have a lot to do with the suppression NLRP3 inflammasome, nuclear factor-kappa B, and phosphatidylinositol 3 kinase/AKT (protein kinase B) signaling pathway activation [58]. Of note, galangin did not exert any cytotoxicity at effective concentrations. This suggests that galangin might be an effective and safe agent for treating hyperuricemia-induced renal damage and that urate-lowering therapy is not the only way [58].

## 6. Conclusion

In summary, this paper surveys experimental research that TCM, in simples or formulas, could mitigate hyperuricemia, via playing a different role in the liver, kidney, and intestine. TCM, like most botanical medicines, contains a variety of active constituents, which act as anti-inflammatory, antioxidation, regulating uric acid transporter, and so on. Many of these herbs work through multiple modes of activity (MOAs) and multiple constituents, more than just a single active constituent hidden in a phytochemical matrix. Conventional western approaches utilizing anti-inflammatory agents with xanthine dehydrogenase and XOD inhibitors could be supplemented with natural drugs in therapeutic cocktails. These observations urge the wider application of TCM in treatment protocols for hyperuricemia. In fact, to able to be more scientific and persuasive, there are things to be done. Firstly, establishment for hyperuricemia model should be standardized. A proper hyperuricemia mouse model asks for a stable, consistent, and high serum urate concentration, while sometimes it is not easy to determine the hyperuricemia for mice. Secondly, qualification control of TCM might as well be uniformed and the adoption of TCM, such as origins and storage, means of administration, and so on. Thirdly, evaluation of safety of mouse research should be involved, say, acute toxicity tests and long-term toxicological tests of herbs, for example, the injuries of kidney. All in all, there is still a long way to go.

## Figures and Tables

**Table 1:** Roles of TCM in the liver, kidney, and intestine to ameliorate hyperuricemia in experimental studies.

Name of herb(s) or formulas	Main ingredient(s) or component(s)	Animal Model(s) and/or cell(s)	Main action(s) and possible mechanism	References
Hepatic effects (in simples)				
*Cinnamomum cassia*	Cassia oil	PO-induced HUA mice	Lower liver XDH/XOD activities	[13, 14]
*Cinnamomum osmophloeum* Kaneh. leaf	Essential oil (cinnamaldehyde)	PO-induced HUA mice	Inhibitory effects on XOD	[15]
*Lonicera hypoglauca* Miq.	Lonicera flavone	In vivo and in vitro PO-induced HUA mice	Suppress XOD activity	[16]
Rhizomes of *Curcuma longa* L.	Curcumin	PO-induced HUA mice	Suppress serum and liver XOD; suppress NLRP3	[17]
*Smilax china* L.	Engeletin, astilbin, polydatin, chlorogenic acid, protocatechuic aldehyde, caffeic acid, rutin, oxyresveratrol, and resveratrol	In vivo and in intro, Fructose induced HUA rats	Suppress XOD activity; inhibiting TGF-*β*1, CTGF, PGE2, IL-1	[18, 19]
Aurantii fructus immaturus carbonisata	Carbon dots	In vivo and in intro, PO & hypoxanthine induced HUA rats and MSU induced gout rats	Inhibit serum and liver XOD activity	[20]
*Cordyceps militaris* (L. ex Fr.) Link	Exopolysaccharides	PO-induced HUA mice	XOD inhibitory activities; renal protection	[21]

Hepatic effects (in formulas)				
Ermiao wan	Phellodendri cortex and rhizome atractylodis	PO-induced HUA mice	Inhibiting XDH and XO activities; improved kidney fibrosis	[22]

Renal actions (in simples)				
*Cordyceps militaris* (L. ex Fr.) Link.	LYS145, ARG477, ARG325, and ASP168	PO-induced HUA mice	Inhibition activity on URAT1 protein levels	[23]
*Herbaceous peony*	Total glucosides	Adenine and ethambutol induced urate	Upregulate renal OAT1, downregulate renal GLUT9 and URAT1; improved kidney histopathological alterations	[24]
Rhizoma Dioscoreae Nipponicae	Total saponins	PO-induced HUA mice	Regulate OAT1, OAT3, URAT1and GLUT9 mRNA and protein levels	[25]
Fraxini Cortex	Aesculetin, aesculin, fraxetin, and fraxin,	Hypoxanthine and PO induce HUA Rats	Downregulated GLUT9 and URAT1 mRNA and protein levels	[26]

Renal actions (in formulas)				
Simiao pill	Phellodendri cortex, atractylodis rhizome, achyranthes root, and coix seed	PO-induced HUA mice	Increase FEUA levels and decreased serum uric acid levels; regulate renal mOAT1, mURAT1, and mGLUT9,renal mOCTN1, mOCTN2, and mOCT1, mOCT2,	[27]
Wuling San	Polyporus umbellatus (Pers.) Fries, Wolfiporia cocos (Schw.) Ryv. & Gibn, Alisma, plantago-aquatica subsp. orientale (Sam.) Sam., Cinnamomi cortex, Atractylodes macrocephala, Koidz.	PO-induced HUA mice and high fructose fed HUA mice	Downregulate renal mGLUT9 and mURAT1 mRNA and protein levels , upregulate those of mOAT1, mOCT1, mOCT2, and mOCTN2;reverse dysregulation of URAT1, GLUT9, ABCG2 and OAT1, and OCT1 and OCT2 ,inhibit TLR4/MyD88 signaling and NF-*κ*B, signaling and MAPKs; suppress NLRP3 and renal secretion of IL-1*β*	[28, 29]
Erding formula (*Erding granule*)	Viola yedoensis Makino Lobelia chinensis Lour, Taraxacum mongolicum Hand.-Mazz. and Isatis, indigotica Fort, PO-induced HUA mice	PO-induced HUA mice	Suppress URAT1 mRNA expression and enhance that of OAT3; analgesic and anti-inflammatory effects; downregulate GLUT9 and URAT1 protein expression and upregulate OAT1 protein expression	[30, 31]
Compound tufuling granules	Rhizoma Smilacis Glabrae, Rhizoma, Dioscoreae Hypoglaucae, Pseudobulbus, Cremastrae seu Pleiones, Semen Vaccariae, Radix Achyranthis Bidentatae	Yeast extract and PO-induced HUA mice	Inhibit GLUT9	[32, 33]
Xie-Zhuo-Chu-Bi-Fang	Smilacis glabrae rhizoma, Heterosmilacis, Rhizoma, Cremastrae Pseudobulbus, Achyranthis, Bidentatae Radix, and Vaccariae Semen	Yeast extract and PO-induced HUA mice	Inhibit URAT1 mRNA expression	[34, 35]
Quzhuotongbi decoction	*Smilax glabra* Roxb., *Dioscorea septemloba* Thunb., Maydis stigma, coix seed, Alismatis rhizome, Humulus scandens, Parasitic loranthus, Herba Siegesbeckiae, turmeric, Corydalis, Rhizoma and Citrus medica	A high yeast-fed HUA rat	Inhibit URAT1 expression	[36]

Renal and intestinal actions (in simples)				
*Dioscorea tokoro* Makino ex Miyabe	Dioscin (tigogenin)	PO-induced HUA mice	Inhibit URAT1and promote ABCG2	[37]
Chicory (*Cichorium intybus* L.)	Aesculin, chicoric acid, chlorogenic acid, 14(15)-diene-3*α*-ol, 13,14-seco-stigma5(6), and isochlorogenic acid A/B/C,	In vivo and in intro, fructose induced HUA mice	Downregulate XOD activity and upregulate renal OAT3 mRNA; downregulate intestinal ABCG2 mRNA and protein. Suppress renal GLUT9 protein	[38–40]

Hepatic and renal dual actions (in simples)				
*Smilax glabra Roxb.*	Total flavonoids (neoastilbin, astilbin, neoisoastilbin, and isoastilbin)	PO-induced HUA mice	Suppress XOD and upregulate the expression of OAT1, OCTN2, and the mRNA (mOAT1 and mOCTN2) in kidney tissue	[41]
*Gnaphalium pensylvanicum* willd.	Caffeoylquinic acid derivatives (chlorogenic acid, 3,5-dicaffeoylquinic acid, 4,5-dicaffeoylquinic acid methyl ester and 1,5- dicaffeoylquinic acid)	PO-induced HUA mice	Downregulate GLUT9 and URAT1 expression, upregulate OAT1 expression, and inhibit XOD activity	[42]
*Smilax riparia*	Total saponins (smilax-chinoside A and smilax-chinoside C. timosaponin J and riparoside B)	PO-induced HUA mice	Downregulate renal mURAT1, inhibit XOD	[43–46]
*Morus alba* L.	Morin(3,5,7,2',4'-pentahydroxyflavone)	In vivo and in vitro, PO-induced HUA mice	Regulate URAT1 (need further research), inhibition of XOD	[47]
*Paederia scandens*	Asperuloside, paederoside, and scandoside	In vivo and in intro, PO-induced HUA mice	Inhibit XOD, inhibit renal urate reabsorption	[48]
*Camellia sinensis* Theaceae	Green tea polyphenols (catechins)	PO-induced HUA mice	Inhibit serum and liver XOD, decrease URAT1 expression and promote OAT1 and OAT3 expression	[49]
Alismatis rhizoma and rhizoma smilacis glabrae	Not available	PO & adenine induced HUA rats	Inhibit XOD, downregulate renal URAT1 expression	[50]

Hepatic and renal dual actions (in formulas)				
Sanmiao wan	Achyranthes root, phellodendri cortex, and atractylodes rhizome	PO-induced HUA mice	Inhibit hepatic XOD, downregulate kidney mURAT1	[51]
Modified Chuanhu anti-gout mixture	*D. nipponica*, *P. cuspidatum*, honeysuckle rattan, Radix cyathulae, glabrous greenbrier, Radix saposhnikoviae, Radix clematidis, Rhizoma chuanxiong, coix seed, Glycyrrhizae radix et rhizome	PO-induced HUA mice	Inhibit liver XOD and kidney URAT1 levels	[52]
ShiZhiFang	Plantago seeds, white mustard seeds, vaccaria seeds, and abutilon seeds	PO-induced HUA rats	Facilitate renal rOAT3 and rOAT1 transcription and expression and inhibit serum XOD activity	[53]

Hepatic, renal, and intestinal triple actions (in simples)				
*Dendrobium officinalis* six nostrum	SiMiao Wan by adding *Dendrobium officinalis*	PO & high-fat sorghum induced HUA with hyperlipidemia rat	Upregulate HPRT1, inhibit liver XOD, increase renal and intestinal ABCG2 content, reduce PDZK1content, and inhibit NLRP3 and caspase 1 activation	[54]

Antioxidative and anti-inflammatory effects				
Rhizoma smilacis glabrae	Water extract	Monosodium urate and PO-induced HUA and gout murine	Upregulating the expression of catalase	[55–57]
*Alpinia officinarum* Hance	Galangin	In vitro	Suppression NLRP3, NF-*κ*B and PI3K /AKT PKB signaling pathway activation	[58]

HUA, hyperuricemia; PO, potassium oxonate; MSU, monosodium urate; XO/XOD, xanthine oxidase; XDH, xanthine dehydrogenase; CTGF, connective tissue growth factor; PGE2, prostaglandin E2; IL, interleukin; URAT1, urate transporter one; GLUT9, glucose transporter nine; OAT1/3, organic anion transporter one and three; FEUA, fraction excretion of uric acid; OCT1/2, organic cation transporter; OCTN1/2, organic cation/carnitine transporter one and two; TLR4/MyD88, toll-like receptor 4 /myeloid differentiation factor 88; NF-*κ*B, nuclear factor-kappa B; MAPKs, mitogen-activated protein kinases; NLRP3, the NOD-like receptor P3 inflammasome; ABCG2, adenosine triphosphate- (ATP-) binding cassette transporter two; HGPRT, hypoxanthine guanine phosphoribosyl transferase; PI3K, phosphatidylinositol 3 kinase; PKB, protein kinase B; PDZK1, PDZ domain protein kidney one; SOD, superoxide dismutase; GSH glutathione; MDA, malondialdehyde.

## Data Availability

No data, code, or models were utilized or generated during the study.
